# P242: Benchmarking of infection related to health care indices: 14 Brazilian health intensive care pediatric and neonatal units

**DOI:** 10.1186/2047-2994-2-S1-P242

**Published:** 2013-06-20

**Authors:** C Silva, EA Bussotti, K Cristensen, AC Alves, LM Ramos Filho, SHF Mendonça

**Affiliations:** 1 RS, Hospital Samaritano, Sao Paulo, Brazil

## Introduction

Infections Related to Health Care (IRHC) today are considered as a public health problem worldwide, therefore it is essential that institutions focus their efforts on the continuous improvement of quality of care using the IRHC indices as indicators of quality, validated and recognized for this purpose, but in Brazil the comparison of IRHC (benchmarking) with the purpose of evaluation is arduous task, since there is no national database for external comparisons, is even scarcer such information in pediatrics and neonatology.

## Objectives

To compare rates of IRHC among intensive care units (ICU) neonatal and pediatric, to improve the quality of care and support for the proposed construction of new national public policies.

## Methods

Prospective cohort study, longitudinal, September 2012 and partial evaluation in December 2012, and the data were grouped into two distinct groups (05 pediatric ICU and 09 neonatal ICU), with distinct purpose of comparisons.

## Results

The distribution of hospitals according to geographic region of the country: 50% North, Northeast 42%, 8% Midwest, according to the type of ICU: 67% Neonatal, Pediatric 33%. The average density Incidence of Primary Bloodstream Infection / 1000 CVC days in the group of Pediatrics ranged from 3.82 to 34.13 and Neonatology group ranged from 2.58 (in the weight range E) to 164 6 (in the weight range D) and the average density of Pneumonia Incidence / 1000 days of MV in the group of Pediatrics ranged from 5.32 to 24 and Group of Neonatology ranged from 3.42 (in the weight range C) at 194 (the weight range B), whereas the density of Urinary Tract Infection / 1000 SVD days ranged from 4.40 to 23.65 (Pediatrics) and 0 in all weight ranges in the Group of Neonatology 11.78 weight in the range of E; density Enterocolitis in neonatology group ranged from 0.17 (weight range D) to 3.24 (the weight range D).

## Conclusion

This study has pointed to the possibility of knowing the reality of care in the Pediatric ICU and Neonatal in Brazil, through indicators that monitor important adverse events such as infection, even in the face of different realities within a country of continental dimensions and not always homogeneous.

## Disclosure of interest

None declared

